# Linking Life Cycle and Integrated Assessment Modeling to Evaluate Technologies in an Evolving System Context: A Power-to-Hydrogen Case Study for the United States

**DOI:** 10.1021/acs.est.2c04246

**Published:** 2023-02-01

**Authors:** Patrick Lamers, Tapajyoti Ghosh, Shubhankar Upasani, Romain Sacchi, Vassilis Daioglou

**Affiliations:** † Strategic Energy Analysis Center, National Renewable Energy Laboratory, Golden, Colorado 80401, United States; ‡ Technology Assessment, Paul Scherrer Institute, 5232 Villigen, Switzerland; § PBL Netherlands Environmental Assessment Agency, 2594 AV The Hague, the Netherlands; ∥ Copernicus Institute, Utrecht University, 3508 TC Utrecht, the Netherlands

**Keywords:** prospective life cycle assessment, integrated assessment modeling, power-to-X, decarbonization, hydrogen, open-source code, LiAISON

## Abstract

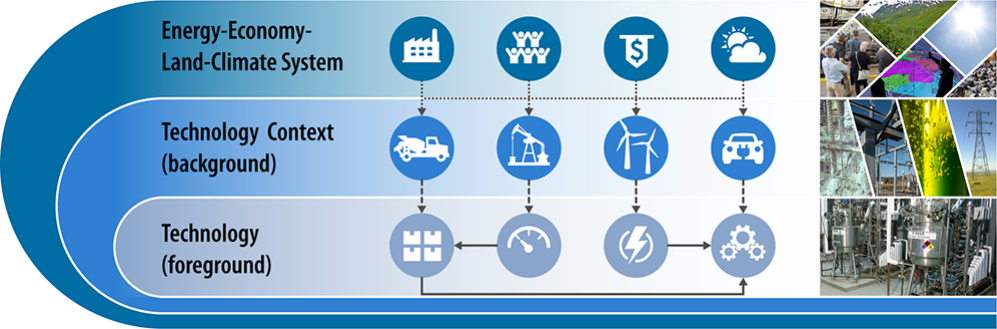

Carbon-neutral hydrogen (H_2_) can reduce emissions from hard-to-electrify sectors and contribute to a net-zero greenhouse gas economy by 2050. Power-to-hydrogen (PtH_2_) technologies based on clean electricity can provide such H_2_, yet their carbon intensities alone do not provide sufficient basis to judge their potential contribution to a sustainable and just energy transition. Introducing a prospective life cycle assessment framework to decipher the non-linear relationships between future technology and energy system dynamics over time, we showcase its relevance to inform research, development, demonstration, and deployment by comparing two PtH_2_ technologies to steam methane reforming (SMR) across a series of environmental and resource-use metrics. We find that the system transitions in the power, cement, steel, and fuel sectors move impacts for both PtH_2_ technologies to equal or lower levels by 2100 compared to 2020 per kg of H_2_ except for metal depletion. The decarbonization of the United States power sector by 2035 allows PtH_2_ to reach parity with SMR at 10 kg of CO_2e_/kg H_2_ between 2030 and 2050. Updated H_2_ radiative forcing and leakage levels only marginally affect these results. Biomass carbon removal and storage power technologies enable carbon-negative H_2_ after 2040 at about −15 kg of CO_2e_/kg H_2_. Still, both PtH_2_ processes exhibit higher impacts across most other metrics, some of which are worsened by the decarbonization of the power sector. Observed increases in metal depletion and eco- and human toxicity levels can be reduced via PtH_2_ energy and material use efficiency improvements, but the power sector decarbonization routes also warrant further review and cradle-to-grave assessments to show tradeoffs from a systems perspective.

## Abstract 2

We introduce a versatile, open-source coded framework that applies structurally consistent background scenarios to assess technologies across metrics over long time horizons to inform decision-making under uncertainty.

## 1 Introduction

The United States (U.S.) government’s ambition of a net-zero greenhouse gas (GHG) emissions economy by 2050^1^ is in line with the Paris Agreement, i.e., a global climate change mitigation target of achieving a maximum average temperature change potential of 1.5 °C or less by 2100 with respect to preindustrial levels.^2^ Achieving the domestic mid-century target will require an accelerated deployment of energy-conserving technologies; a decarbonization of the power and transport sectors via electrification, fuel switching, and expansion of variable renewable energy sources and storage technologies; and increased electrification of the buildings and industrial sectors.^3^ Power and transportation sectors account for the largest sector contributions to total U.S. national GHG emissions with 29 and 25%, respectively.^4^ Their decarbonization routes have been described and modeled extensively;^5,6^ still, the power sector’s scale and the transport sector’s heterogeneity will require a concerted effort to deploy respective strategies and achieve 2035 and 2050 targets accordingly. The industrial sector, accounting for 23% of total U.S. GHG emissions,^4^ is represented by a number of hard-to-electrify activities. These activities require technology solutions that are far less understood or have yet to be scaled. The chemicals subsector has the single largest subsector emissions profile after direct emissions from fossil fuel combustion and leakage from fossil fuel distribution systems.^4^ Within the chemicals sector, many processes depend on hydrogen or ammonia precursors. Decarbonizing these two commodities would contribute significantly to decarbonizing the industrial sector, as hydrogen can also be used for low-carbon steel production (e.g., hydrogen-based direct reduction of iron) and other industrial applications.

Emerging technologies require the application of prospective life cycle assessment (LCA),^7^ which can account for technology (foreground) scaling and process improvements via learning by doing, among others. In many cases, the future system context (background) in which the technologies are assumed to operate is equally relevant.^8^ Background scenarios generated by integrated assessment models (IAMs) can coherently incorporate future dynamics of the energy-economy-land-climate system. Furthermore, IAM scenarios are harmonized across socioeconomic and climate change mitigation pathways,^9^ which facilitates the comparability of prospective LCAs using different IAMs.

Here, we introduce an open-source prospective LCA framework, the Life-cycle Assessment Integration into Scalable Open-source Numerical models (LiAISON), to analyze the non-linear relationships between technology foreground and the future energy system background across a series of midpoint and resource-use metrics. We showcase LiAISON by assessing two power-to-hydrogen (PtH_2_) processes, namely, solid oxide electrolysis (SOE) and polymer electrolyte membrane electrolysis (PEME). We compare the technologies to a baseline of hydrogen production via natural gas-based steam methane reforming (SMR) without carbon capture and storage (CCS) in a U.S. context of multiple-energy system and climate change mitigation futures. Using high-performance computing, we specify the impact of background and foreground dynamics on the results. As well as providing an analysis that specifies the LCA result ranges with temporal and geospatial explicitness across the two technologies, metrics, and impact assessment methods, this paper also aims to establish a base framework that can be expanded to use other IAM-generated scenarios and U.S. open-source life cycle inventory (LCI) databases.

## 2 Materials and Methods

**Figure 1:**
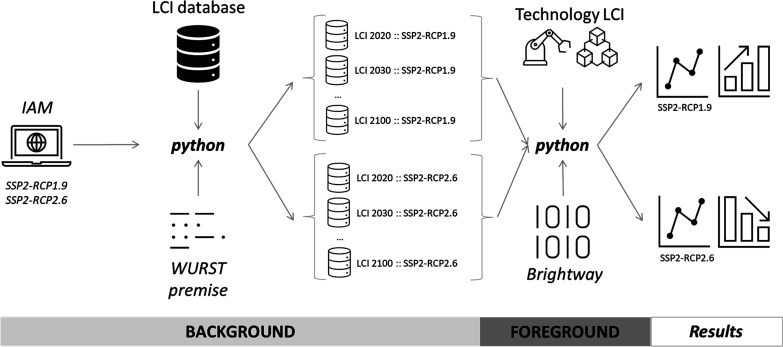
LiAISON framework schematic.

LiAISON consists of multiple components, which are linked via a Python-coded script (Figure 1). The first step in the framework is the systematic modification of LCI databases with external scenario data. Using the library *premise*,^10^ the code processes IAM scenarios to modify the original LCI database^11^ and creates scenario-specific time-step database images. The resulting database images account for scenario-specific changes in technologies, related emissions, and supply chains and represent comprehensive backgrounds, which feed the prospective technology assessments. The library produces these scenario-specific database images by changing the material and energy efficiency of processes contained in the LCI database, emissions, and relative shares of market inputs and separating global market data sets into region-specific ones. Adaptations include code updates for enhanced computational efficiency, the addition of a stochastics element, and LCI data revisions. The LCIs for the technologies in focus were compiled separately. LiAISON automatically reads tabular file LCIs and uses the LCA library *Brightway*(12) to compute the midpoints and resource-use impacts per technology and scenario for every year within the simulation.

### 2.1 Models

The future energy system scenarios were derived from the IAM *IMAGE 3.2*,^13,14^ which describes the relationships between humans and natural systems and the impacts of these relationships on the provision of ecosystem services to sustain human development. Its energy module *TIMER* is a recursive dynamic energy system model representing the global energy system, disaggregated across 26 global regions, with projections toward 2100.^13^ It includes fossil and renewable primary energy carriers (coal, heavy/light oil, natural gas, modern/traditional biomass, nuclear, concentrated/photovoltaic solar, onshore/offshore wind, hydropower, and geothermal). Primary energy carriers can be converted to secondary and final energy carriers (solids, liquids, electricity, hydrogen, heat) to provide energy services for different end-use sectors (heavy industry, transport, residential, services, chemicals, and others). The model projects future (useful) energy demand for each end-use sector based on relationships between energy services and activity, the latter of which is related to economic growth and endogenous developments in energy prices. For each demand sector, secondary energy carriers (including solid and liquid biofuels) compete for market shares to meet the useful energy demand, based on relative costs (including capital and variable costs), where the cheapest option gets the largest market share. The model thus does not follow a purely optimization solution. Projected energy prices are based on supply curves of energy carriers.^15,16^ Non-renewable sources are formulated in terms of cumulative extraction, while for renewable sources, these are formulated in terms of annual production.^17−19^*Brightway*^12^ is an open-source framework for LCA calculations in Python consisting of several modules that handle importing data, managing and accessing data, calculating, and analyzing LCA results. It also contains various characterization methods. *Brightway* reads the scenario-specific LCI databases produced by *premise*(10) to calculate life-cycle midpoint environmental indicators and resource uses. The main findings apply the ReCiPe^20^ characterization method due to its choice of indicators for a holistic sustainability assessment and because it has been updated more recently than TRACI.^21^ For completeness, we also apply TRACI, with results provided in the Supporting Information.

### 2.2 Background Scenarios and Dynamics

The library *premise* is given climate change mitigation scenarios developed by IMAGE to alter the background LCI data of our prospective LCA. IMAGE scenarios are built as combinations between narratives of the Shared Socioeconomic Pathways (SSPs)^9,22^ and climate targets defined by the Representative Concentration Pathways (RCPs).^23^ A key benefit of applying these scenario combinations is that they are “standardized” outputs for IAMs, allowing comparisons across models and interchangeability of inputs. Thus, the technology assessment is performed in an integrated systems context that is widely used to compare and evaluate different climate change mitigation pathways—as reported, for instance, by the Intergovernmental Panel on Climate Change (IPCC).^24^

We apply a “Middle of the Road” socioeconomic pathway (*SSP2*), assuming future demographic, economic, technological, and behavioral developments that are in line with historical patterns. The reference scenario (*SSP2-baseline*) does not consider any climate policies and measures to limit radiative forcing or to enhance adaptive capacity. Given the SSP2 socioeconomic pathway, an appropriate carbon price is endogenously calculated to ensure that specific RCPs are met; in this case, RCP1.9 and RCP2.6. These scenarios signify radiative forcing levels of 1.9 and 2.6 W/m^2^, respectively, or a global mean surface temperature increase of 1.5 and 2 °C by 2100 relative to pre-industrial levels, respectively. Thus, these “mitigation” scenarios are aligned with achieving the Paris Agreement objectives.

The background LCI data dynamics represent changing sector structures for electricity, cement, steel, and fuels. Updating the electricity inventories implies an alignment of regional electricity production mixes as well as efficiencies for several electricity production technologies, including CCS technologies and photovoltaic (PV) panels. The update of the inventories for cement (with optional CCS) includes an adjustment of technologies for cement production (dry, semi-dry, wet, with pre-heater or not), fuel efficiency of kilns, fuel mix of kilns (including biomass and waste fuels), and clinker-to-cement ratio. The steel industry is represented by primary and secondary production routes, using blast furnace–basic oxygen furnace (BF-BOF) and electric arc furnace (EAF), respectively. The code adjusts the process efficiency and fuel mix of the BF-BOF route and adds post-combustion, amine-based CCS if necessary, while the EAF benefits from the decarbonization of the electricity sector in the region. The *premise* library also corrects the supply shares from BF-BOF and EAF in the regional steel market, as indicated by the IMAGE scenario. Fuel background changes include the creation of regional markets for liquid and gaseous fuels and relinking fuel-consuming activities.

### 2.3 Foreground Calibration and Dynamics

The functional unit of the technology assessment is 1 kg of hydrogen (H_2_). We compare two processes to the standard production via SMR from natural gas (Figure S1). SMR is the predominant process to produce hydrogen in the United States. Apart from the consumption of methane, the reaction produces 1 mol of carbon dioxide for every 4 mol of hydrogen. Attractive decarbonization routes include those that produce hydrogen by splitting water molecules using electrolysis, such as SOE and PEME. PEME uses a proton exchange membrane made from a solid polymer electrolyte to conduct protons from the anode to the cathode, resulting in the electrolysis of water to create hydrogen and oxygen gases (Figure S2). The SOE process uses a fuel cell made of a solid oxide electrolyte that conducts negative oxygen ions from the cathode to the anode, resulting in water splitting (Figure S3). The underlying LCI data for PEME and SOE are literature-based,^25,26^ reflecting scales of 150 kW_el_ for SOE and 1 MW_el_ for PEME.

In prospective LCA, potential future technology improvements need to be accounted for, which influence material and energy use efficiencies. Often, such improvements are approximated in LCA using learning curves, depicting the cost reductions per unit over time. Multi-factor learning curves are used to capture unit cost improvements through all technology readiness levels. Single-factor learning curves limit improvements to the deployment and learning-by-doing stage.^27−30^ Applying the single-factor learning curve seems appropriate at the scale of this analysis, since the assessed technologies are deployed in a long-term scenario context. The basic principle of the single-factor learning curve is that with each doubling of cumulative production, the cost per unit drops by a learning parameter *b.* The resulting learning rate (LR) is expressed via the following formula:
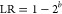


Peer-reviewed empirical data shows the LR for PtH_2_ technologies to be 18%,^31,32^ which is also the value applied in IMAGE. Yet, these studies are only partially based on PEME systems and it is unclear whether this LR would apply to similar, yet different electrolysis-based technologies like SOE. Furthermore, LRs are usually calculated based on capital rather than production costs and it can be debated whether unit cost reductions directly translate into material and energy efficiency improvements. Here, we opted for a more conservative LR of 5% per doubling of cumulative production to determine *b*. The LR is kept the same across scenarios but varied between 1 and 10% in a sensitivity analysis to assess the relative importance of background vs background plus foreground dynamics. LRs are typically found to be constant^27−29^ over time (log linear), an observation we adopted for this analysis.

The LR allows us to derive the learning parameter *b*, which was used to translate unit cost reductions into energy and material use efficiency improvements using the following formula:
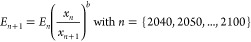
where *E_n_* is the efficiency parameter at scenario timestep *n* (e.g., 2040) and *E*_*n*+1_ is the efficiency parameter at the following scenario timestep (e.g., 2050). *x_n_* is the cumulative flow of material in scenario timestep *n*, and *x*_*n*+1_ is the cumulative flow of material in the following scenario timestep. The baseline lower heating value efficiencies (*E*_2040_) per technology are set to 60% for PEME, 63% for SOE, and 73% for SMR.^26,33^ We capped the efficiencies for all technologies at 93% to remain within thermodynamic limits. The incumbent or reference technology SMR (without CCS) was not given an LR. The assumption of a static reference with a CCS option can be debated. Yet, we also did not account for varying methane leakage rates, a factor that would likely impact the results for SMR. The U.S. natural gas supply mix is based on the respective LCI database^11^ entry, which assumes that 90% is domestically produced with roughly 10% being imported from Canada and Mexico (on an energy basis). Of the domestic production, 70% is generated by dedicated natural gas and the remainder at oil and gas extraction sites. All extraction was assumed to occur onshore. The methane leakage rate of this inventory is 1.33% of the mass of natural gas distributed (e.g., 13.3 g CH_4_/kg) representing 65% of the global warming impact from natural gas extraction and supply.

## 3 Results and Discussion

**Figure 2:**
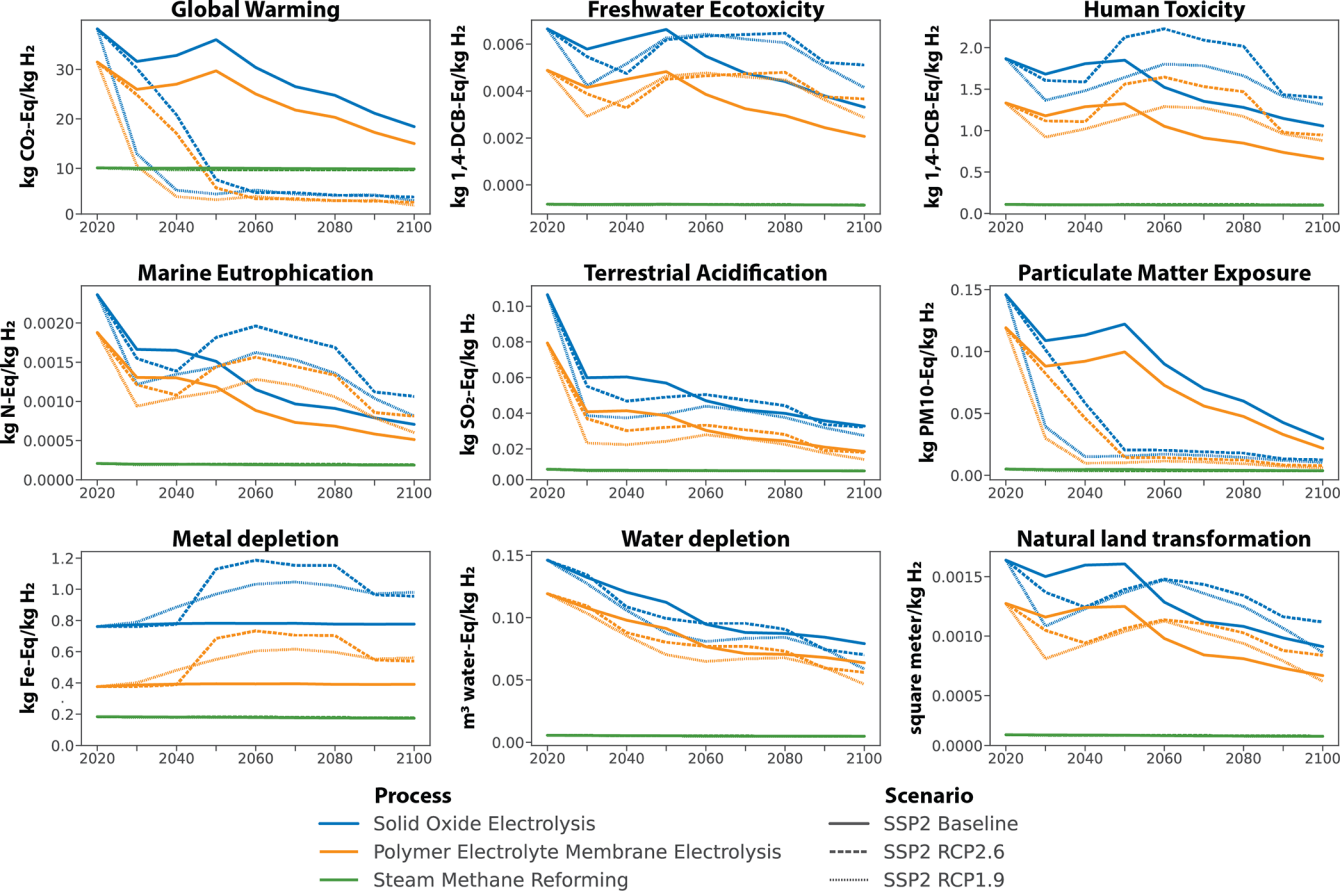
Comparison of all technologies across scenarios under a changing U.S. multisector context (background dynamics only).

**Figure 3:**
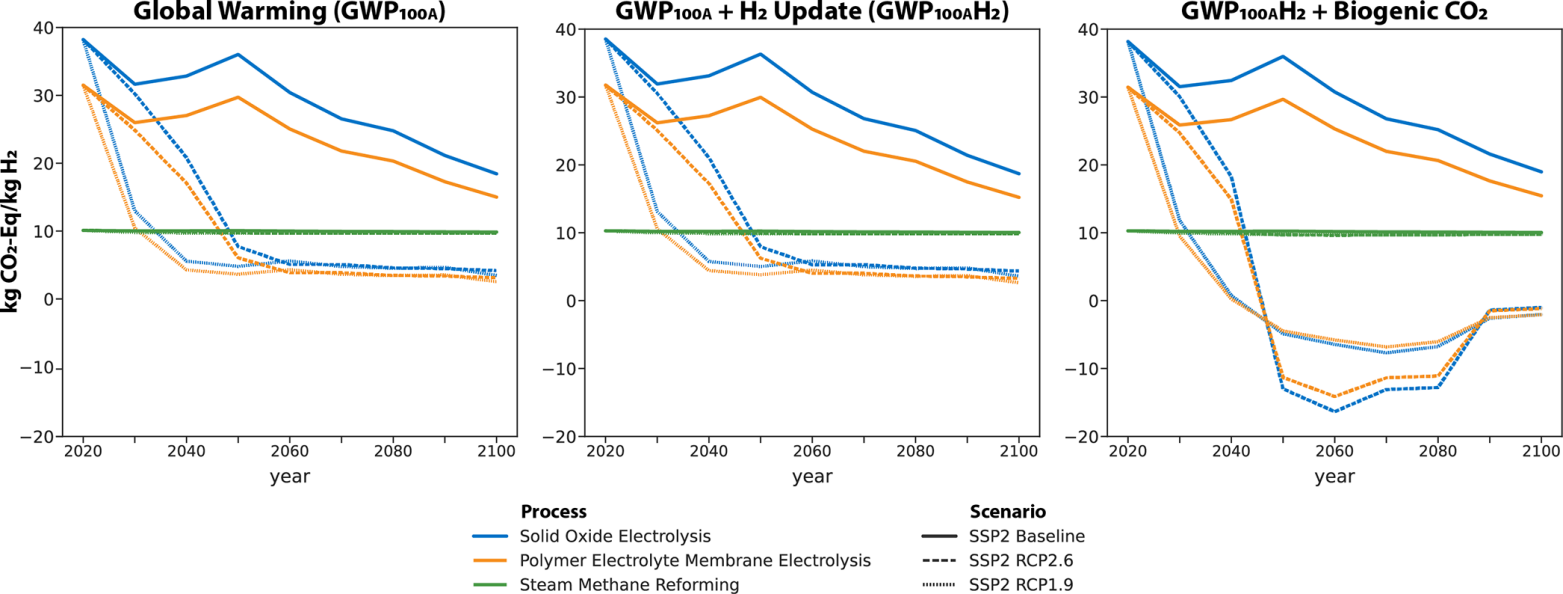
Global warming effects using the default ReCiPe method GWP_100_ (left panel), considering GWP_100_ with an updated radiative forcing level for hydrogen (middle panel), and accounting of biogenic CO_2_ as carbon-neutral or negative if CCS is applied (right panel).

Comparing the two PtH_2_ technologies against the SMR baseline in a dynamic U.S. system context (i.e., changing sector structures for power, cement, steel, and fuels) shows variations and dependencies over time (Figure 2). Overall, the PEME process shows lower environmental impacts than SOE, a fact that is likely influenced by the smaller system scale. The temporal environmental performance of either technology and their difference to SMR is directly influenced by the underlying background dynamics. Under baseline projections (i.e., no decarbonization goals), neither electrolysis process reaches parity with the incumbent technology across the observed metrics (Figure 2). Under the decarbonization scenarios, the underlying sectoral shifts result in declining impacts over time compared to 2020 levels, except for metal depletion levels, which increase. The background shifts postulate a heavily decarbonized economy and energy system, which facilitates that technologies reach parity to SMR between 2040 and 2050 (RCP2.6) and between 2030 and 2040 (RCP1.9) for global warming. The reference technology level of 10 kg of carbon dioxide equivalent per kg of hydrogen (kg CO_2e_/kg H_2_) falls within the range of recent estimates of 9–12 kg of CO_2e_/kg H_2_ for SMR.^34^ The specific point in time when the PtH_2_ technologies will reach parity to SMR for global warming will further depend on process configurations such as the addition of CCS technologies and assumed methane leakage rates. The general timeline for parity with respect to global warming between 2030 and 2050 still holds considering a higher radiative forcing level for hydrogen, based on recent respective concerns and discussions.^35^ Yet, if we account for biogenic CO_2_ emissions in the power sector as carbon-neutral or carbon-negative when combined with CCS, postulating that the biomass used was additional, i.e., purpose-grown for energy and thus absorbed CO_2_ from the atmosphere during photosynthesis, a process that would not have occurred otherwise, the two processes can provide carbon-negative H_2_ after 2040 for as low as −14 kg CO_2e_/kg H_2_ for PEME and −16 kg CO_2e_/kg H_2_ for SOE by 2060 (Figure 3). Note that we did not model the potential feedback effects of providing a respective carbon-negative fuel to the energy system in IMAGE, which may have changed the composition of the sectors’ technology portfolios in the decarbonization scenarios.

Despite declines across most other metrics over time, neither PtH_2_ technology can break even with SMR by 2100 besides for global warming. We observe that the reductions in carbon emissions support a constant reduction of acidification impacts (Figure 2). The drop in particulate matter exposure can be directly attributed to the phaseout of fossil fuel combustion across the background mitigation scenarios. Water depletion levels decrease over time as the expansion of energy generation technologies without cooling needs outpaces those with additional water requirements, e.g., for growing bioenergy feedstock. Several metrics exhibit non-linear trends, including freshwater ecotoxicity, human toxicity, marine eutrophication, and natural land transformation. These trends correlate with future deployment levels of specific energy generation technologies—natural gas with CCS and bioenergy with carbon capture and storage (BECCS), primarily—as outlined in the following sections.

### 3.1 Background Dynamics across Scenarios

**Figure 4:**
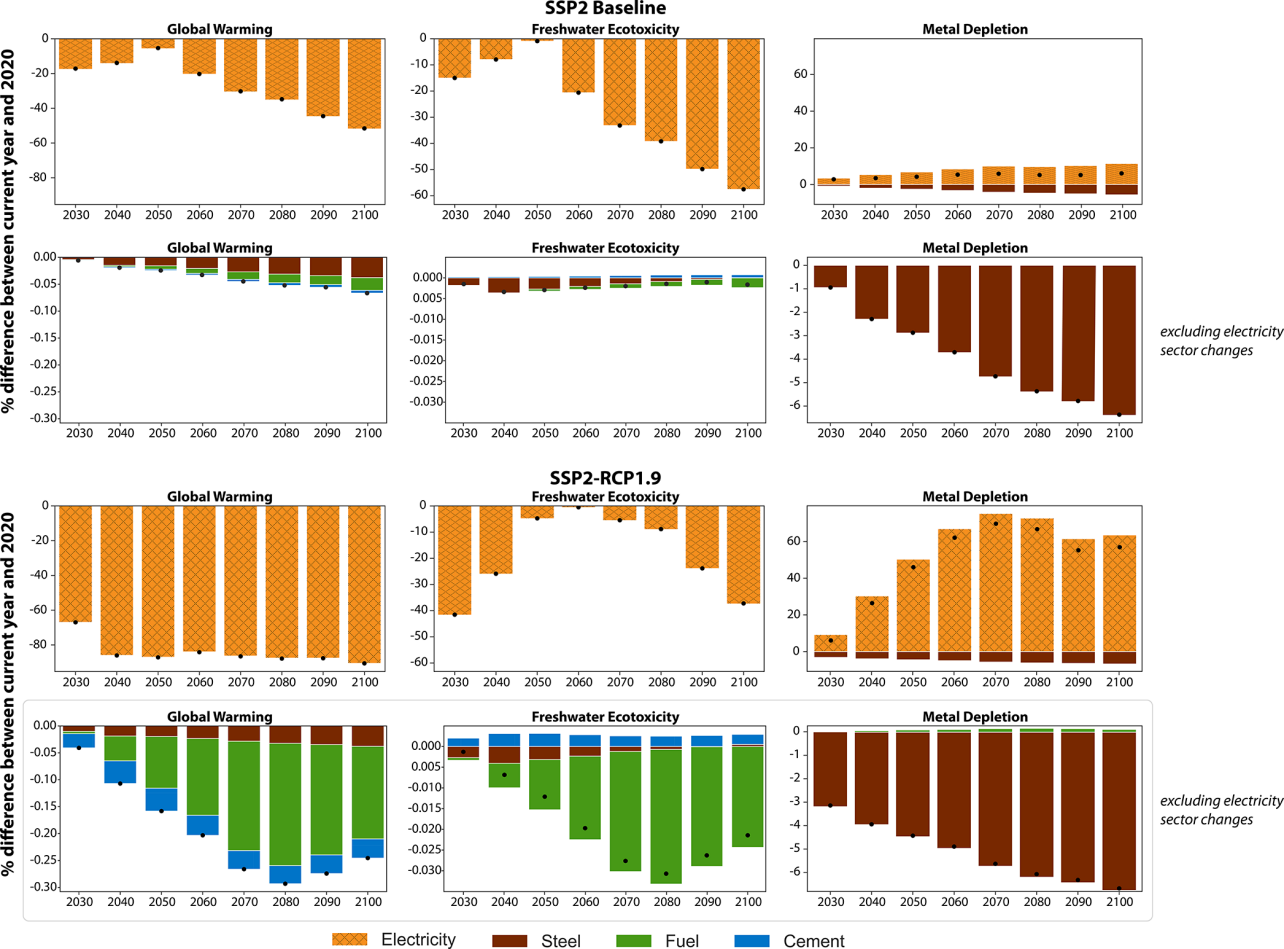
Stacked effects of four background sector dynamics in the SSP2-Baseline (top two rows) and SSP2-RCP1.9 (bottom two rows) scenarios for PEME; points depict net effects.

Breaking out the background dynamics by individual sectoral changes, we find that the PtH_2_ technologies are mainly influenced by, and their impact variations directly correlated with, the changes in the U.S. electricity sector composition (Figure S5). This finding is consistent across all scenarios, metrics, and PtH_2_ technologies. Dynamics within the steel sector also have noticeable effects on metal depletion (i.e., levels are decreased), but the magnitude of the electricity sector effects necessitates a separate view of the sectoral impacts besides power (Figure 4). Across four selected metrics, we find that the changes in the steel sector are relatively consistent between the scenarios and are caused by reduced energy intensity (global warming) and an increase in recycling (ecotoxicity, metal depletion, land transformation) for PEME. Dynamics in the cement and fuel sectors align for global warming and ecotoxicity between the scenarios but trend in opposite directions for natural land transformation. Thus, sectoral background dynamics do not always trend metrics in the same direction and warrant a sector-specific contribution analysis (Figure 4). For instance, the baseline conditions of the fuel sector led to a small increase in natural land transformation, while the fuel sector’s composition in the decarbonization scenario reduces the same metric noticeably. Specifying the background changes per sector also shows reinforcing and counteracting trends per metric over time. The net drop in metal depletion levels in the baseline, as compared to 2020 levels, is facilitated by the changes in the steel sector (i.e., secondary steel production increases, reducing the need for iron ore extraction). The same beneficial changes in the steel sector are outweighed by the more drastic changes in the electricity sector in the decarbonization scenario.

### 3.2 Foreground Dynamics

**Figure 5:**
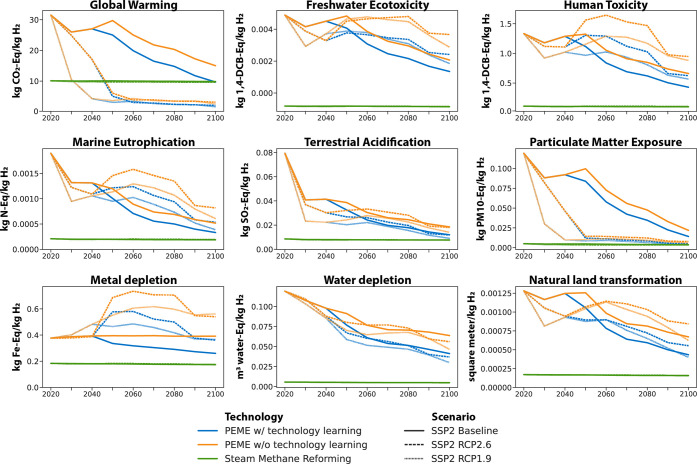
The effects of technology improvements via learning by doing for PEME (foreground dynamics) in addition to background system transformations over time.

Prospective LCA needs to account for potential future technology improvements. In the case of our PtH_2_ technology case study, we account for possible improvements starting in 2040 when electrolysis is deployed globally on a large scale across the background scenarios. The improvements via learning by doing are captured in a learning parameter, which informs material and energy use efficiency improvements between the 10-year scenario timesteps. The effects from learning are described as foreground dynamics, which need to be added to the background dynamics. To evaluate the potential effects of foreground dynamics, we now need to compare the dual dynamics with “background only” dynamics. Figure 5 shows these effects for PEME.

**Figure 6:**
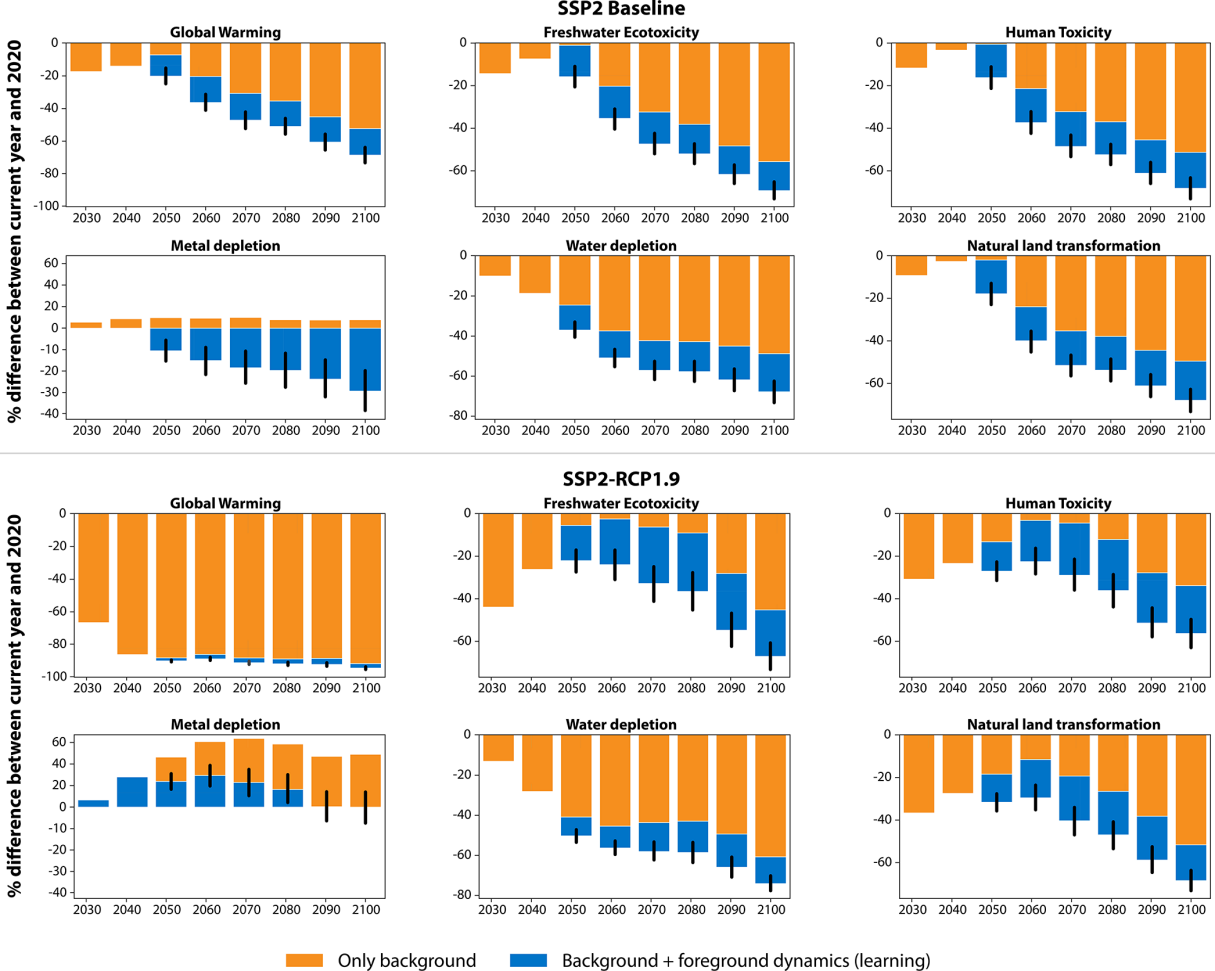
Distinguishing the temporal contributions for selected metrics by background dynamics (only) vs background plus foreground dynamics (additional technology improvements via learning by doing) for PEME across two scenarios.

We find that the additional effects due to large-scale deployment, learning by doing, and efficiency improvements reduce impacts consistently across the technologies, scenarios, and metrics evaluated. Yet, the magnitude of the foreground dynamics for PtH_2_ technologies varies across scenarios and metrics (Figure 5). Technology improvements are relatively more important in scenarios and metrics less affected by background changes. For instance, the additional reductions for global warming due to foreground dynamics critically reduce effects in the baseline but are marginal compared to background changes in the mitigation scenarios. Still, improvements related to learning are important for reducing freshwater ecotoxicity and metal depletion levels in the mitigation scenarios. Thus, technology improvements can compensate or buffer effects driven by background dynamics (Figure 6). The importance of background dynamics is especially noteworthy in scenarios with radical transformations in one or more of the observed sectors (cement, electricity, steel, and transportation fuels). The PtH_2_ technologies’ heavy reliance on electricity makes these technologies very susceptible to the power sector’s technology composition. Tradeoffs between decreasing climate change impacts and other metrics do exist for both PtH_2_ technologies. The tradeoffs can be addressed over time, to some extent, via respective material and energy efficiency improvements. The benefit of these enhancements varies and is greatest for metrics that see a large increase without foreground improvements. Yet, we do observe an effect of diminishing returns for additional improvements via technology learning.

## 4 Discussion

The framework presented herein aims to support the assessment of emerging technologies in future system contexts and provide guidance to research, development, demonstration, and deployment (RDD&D) prioritization and decision support. The tradeoffs found in this technology case study stress the importance of a multi-metric, prospective LCA framework to inform such high-level decision-making and avoid strategies based on a single or limited set of metrics and ultimately potential environmental problem shifting. Particularly, it stresses that a shift to a decarbonized power sector will reduce the environmental effects of power-dependent technologies from a GHG perspective, yet other impacts, specifically ecotoxicity and resource depletion including metal, trend upward over time, suggesting that changes within the power sector decarbonization trajectories are required to alleviate such tradeoffs. Since some metrics will be further influenced by (sub-)regional conditions, e.g., soil types, the results should be regarded as informing trends with GHG emissions and resource depletion parameters being the most critical to consider.

While the framework is versatile and can be used by researchers, decision-makers, and industry, its current version is computationally intensive. The case study was calculated using high-performance computing infrastructure, which is unlikely to be available to most LCA practitioners. The computational load problem was evaluated several times, and adaptations were made for computational efficiency. Still, benefits of this code-based framework include, among others, that it can readily switch between life cycle impact assessment methods. Applying TRACI generates similar (yet not identical) results across midpoints, matching those of ReCiPe (Figure S6). TRACI also provides additional results, e.g., for ozone depletion, a metric not covered by ReCiPe. The main environmental tradeoffs, and ultimately the conclusions of this case study, hold across both methods.

The framework also allows for regionally explicit LCA as it pertains to specific countries and world regions. The case study illustrating the framework’s capabilities was situated in the United States, and regional input factors and local conditions are accounted for. Comparing the U.S. results to operating conditions in Europe and China for the same technologies, background sector shifts, and scenarios, we see a widely varying carbon footprint in the initial years that trends to a harmonized value in the decarbonization scenarios (Figure S7).

### 4.1 Literature Comparison

Prior LCA studies of the two technologies in a U.S. energy system context found global warming impacts of 29.5 kg of CO_2e_/kg H_2_ via PEME and 23.3 kg of CO_2e_/kg H_2_ via SOE, also applying ReCiPe.^36,37^ In comparison, we found 31 kg of CO_2e_/kg H_2_ for PEME and 38 kg of CO_2e_/kg H_2_ for SOE in 2020, which drop to 12.6 kg of CO_2e_/kg H_2_ for PEME and 15.6 kg of CO_2e_/kg H_2_ for SOE in 2100 under an SSP2-RCP1.9 scenario, accounting only for background sector changes and no additional technology learning (foreground). Similar proximities to literature values are observed for other available metrics, except for ozone depletion (Figure S8). A key difference and underlying reason for the better performance of SOE vs PEME in Mehmeti et al.^37^ is the assumed system scale, which is different to our analysis. We based our inventory for SOE on a more comprehensive LCI,^26^ yet at a smaller scale. While both scales are valid, we refrained from scaling SOE to a larger size as scaling effects on LCI data are non-linear. In the end, the variations strongly emphasize that the assumed initial system design is a key determining factor on results.

### 4.2 Limitations and Future Work

Several assumptions and limitations are present across this work. First, it has not yet been empirically observed that LRs for electrolyzers do affect both energy and material use efficiencies, as assumed herein. Methane leakage linked to natural gas supply is another sensitive input that has not been varied across our analysis. Changing to a recent database release with updated U.S.-based natural gas supply LCI data^38^ based on 2019 production and trade statistics would increase the global warming impact of natural gas by 11% and increase the respective indicator by 0.3 kg of CO_2e_ per kg of SMR-based H_2_, leaving the overall conclusions of this study unchanged. The conclusions also hold true under an extreme case of doubling the upstream leakage rates, increasing the global warming impact of 1 kg of natural gas by 80% and increasing the respective impact by 1.7 kg of CO_2e_ to approximately 12 kg of CO_2e_ per kg of SMR-based H_2._

The estimation of pollutant flows and impacts far into the future increases the uncertainty related to specific results. The range of uncertainty likely increases with the projection period. Underlying factors might include foreground technology improvements and background system changes across the economy, as well as technology breakthroughs. Furthermore, there is uncertainty linked to data quality and completeness during LCI compilation. To incorporate the uncertainty related to data inputs, which propagate through the calculations, we plan to quantify the cumulative effect of uncertainty across all input data. An example test case that needs further refinement is provided in the Supporting Information (Figure S9).

Future recycling rates for different metals used (e.g., in solar PV and wind turbines) are left unchanged and thus equal to current rates. This is a common simplification that is also found across other studies.^39,40^ Yet, IAMs, including IMAGE, are starting to improve their representation of material flows, including recycling rates, by modeling stocks of infrastructure. Thus, future versions of LiAISON will be able to dynamically account for such variations.

LiAISON is also currently extended to utilize input scenarios from the U.S. Department of Energy (DOE)-funded Global Change Analysis Model (GCAM),^41^ another well-established IAM. This capability will be linked, via an ongoing collaboration to expand *premise*, the underlying code structure that can differentiate background changes and help determine cross-sectoral effects of decarbonizing the economy. LiAISON is envisioned to eventually be connected to a user-maintained and populated open-source LCI database (e.g., U.S. LCI) to allow user-defined scenario and model inputs (e.g., sector-specific models).

## Supporting Information Available

Figure S1. Steam methane reforming (SMR) process flow diagram and system boundary. Figure S2. Polymer electrolyte membrane electrolysis (PEME) process flow diagram and system boundary. Figure S3. Solid oxide electrolysis (SOE) process flow diagram and system boundary. Figure S4. Global cumulative hydrogen production via electrolysis across scenarios. Figure S5. US electricity mix under baseline, RCP2.6, and RCP1.9 mitigation scenarios for SSP2. Figure S6. Comparison of all technologies across scenarios under a changing US multi-sector context (background dynamics only) when applying TRACI. Figure S7. PEME technology impacts across different world regions in comparison to the USA. Figure S8. Comparison of SSP2-RCP1.9 results to values by Mehmeti et al. and Häfele et al. per kg of H_2_. Figure S9. Stochastics analysis for PEME across three scenarios. Additional results for other technologies, scenarios, impact assessment methods, and so on, are available from the authors upon request (PDF)
