# Supplementary material for: Linking Life Cycle and Integrated Assessment Modeling to Evaluate Technologies in an Evolving System Context: A Power-to-Hydrogen Case Study for the United States
Source: Environ Sci Technol. 2023 Feb 1;57(6):2464–73. doi: 10.1021/acs.est.2c04246 (PMC9933533; doi:10.1021/acs.est.2c04246)
Supplement: Supplementary file 1 — es2c04246_si_001.pdf [file es2c04246_si_001.pdf]

SUPPORTING INFORMATION

Linking life cycle and integrated assessment modeling to evaluate technologies in an evolving system context: a Power-to-Hydrogen case study for the United States

Patrick Lamers[1,*], Tapajyoti Ghosh[1], Shubhankar Upasani[1], Romain Sacchi[2], Vassilis Daioglou[3,4]

1: Strategic Energy Analysis Center, National Renewable Energy Laboratory, Golden, CO 80401, United States
2: Technology Assessment, Paul Scherrer Institute, 5232 Villigen, Switzerland
3: PBL Netherlands Environmental Assessment Agency, 2594 AV The Hague, the Netherlands
4: Copernicus Institute, Utrecht University, 3508 TC Utrecht, the Netherlands
* Corresponding Author: patrick.lamers@nrel.gov

Content

Figure S1. Steam methane reforming (SMR) process flow diagram and system boundary.

Figure S2. Polymer electrolyte membrane electrolysis (PEME) process flow diagram and system boundary.

Figure S3. Solid oxide electrolysis (SOE) process flow diagram and system boundary.

Figure S4. Global cumulative hydrogen production via electrolysis across scenarios.

Figure S5. US electricity mix under a baseline, RCP2.6, and RCP1.9 mitigation scenario for SSP2.

Figure S6. Comparison of all technologies across scenarios under a changing US multi-sector context (background dynamics only) when applying TRACI.

Figure S7. PEME technology impacts across different world regions in comparison to the US.

Figure S8. Comparison of SSP2-RCP1.9 results to values by Mehmeti et al. (2018) and Häfele et al. (2016) per kg H2.

Figure S9. Stochastics analysis for PEME across three scenarios.

Process Flow Diagrams

Steam methane reforming uses high-temperature steam (700°C-1,000°C) to separate hydrogen from a methane source, typically natural gas. The methane reacts with steam under 3-25 bar pressure in the presence of a catalyst to produce hydrogen, carbon monoxide, and a relatively small amount of carbon dioxide. Steam reforming is endothermic, i.e., heat must be supplied to the process for the reaction to proceed. Subsequently, in what is called the 'water-gas shift reaction' the carbon monoxide and steam are reacted using a catalyst to produce carbon dioxide and more hydrogen. In a final process step, pressure-swing adsorption, carbon dioxide and other impurities are removed from the gas stream, leaving essentially pure hydrogen.

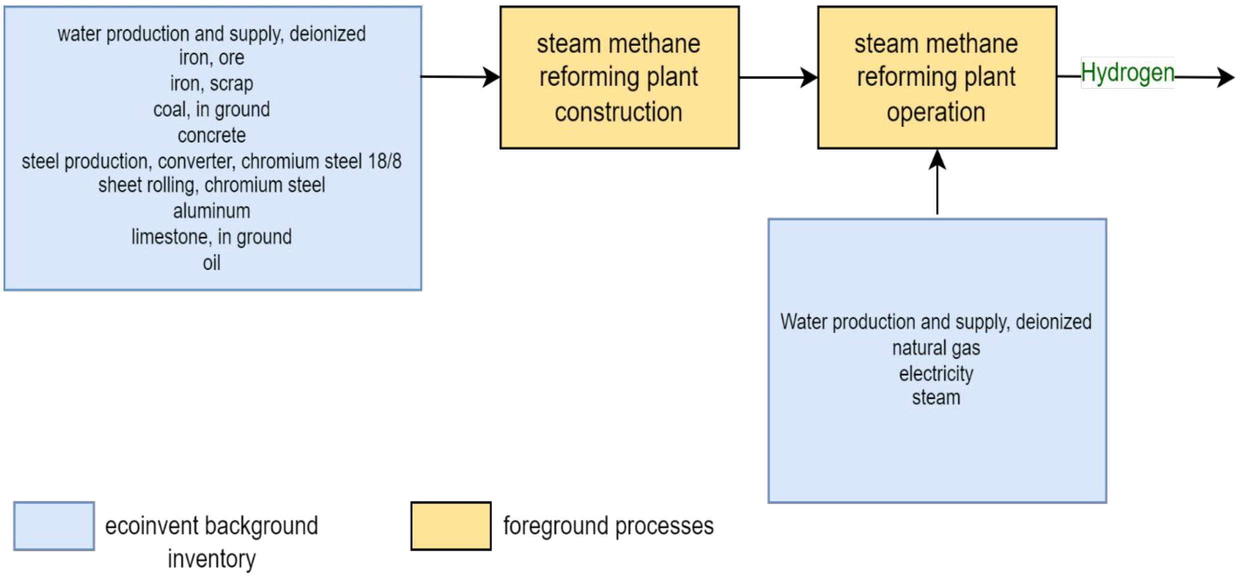

Figure S1. Steam methane reforming (SMR) process flow diagram and system boundary

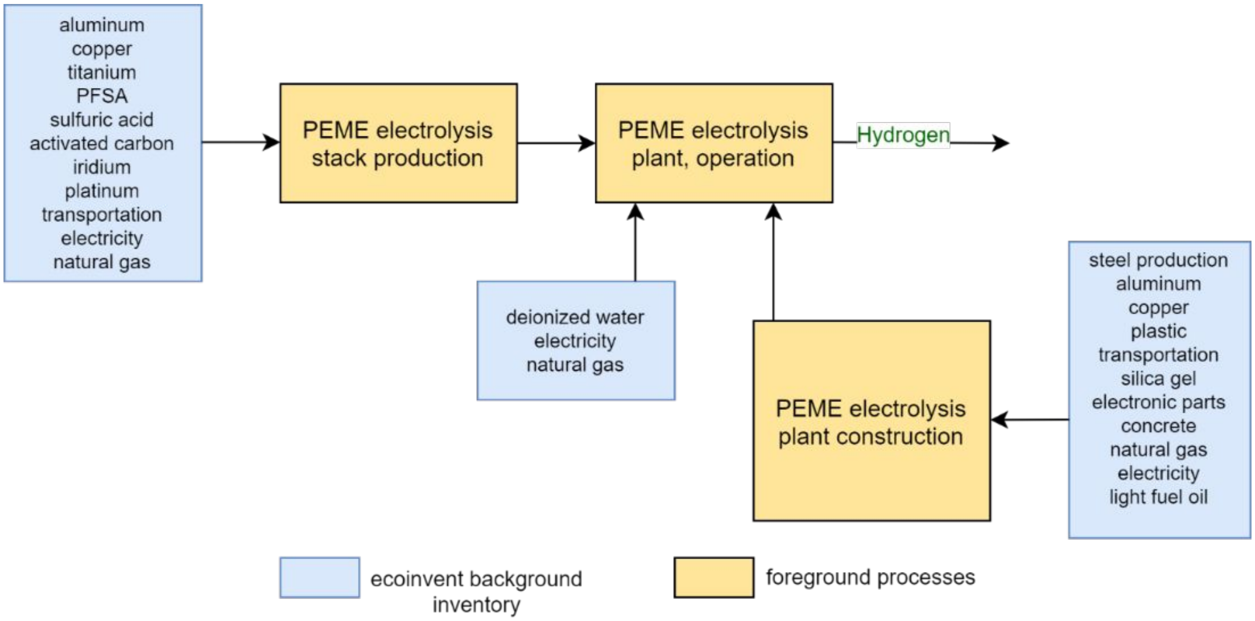

Figure S2. Polymer electrolyte membrane electrolysis (PEME) process flow diagram and system boundary.

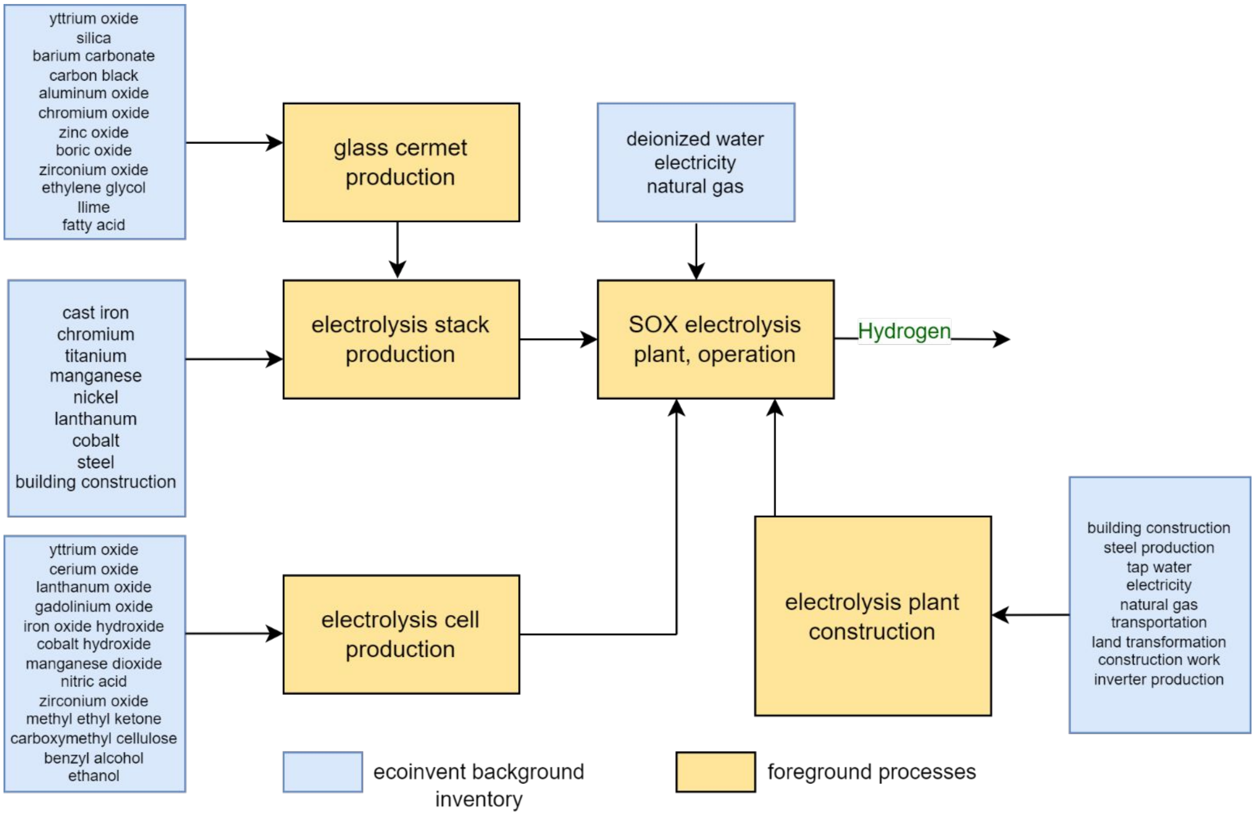

Figure S3. Solid oxide electrolysis (SOE) process flow diagram and system boundary.

Global cumulative H2 production via electrolysis

Table S1. Global cumulative H2 production via electrolysis – projected by IMAGE 3.2.

PJ	**2020**	**2030**	**2040**	**2050**	**2060**	**2070**	**2080**	**2090**	**2100**
**SSP2-Baseline**	0.000001	0.000002	3.78	31.28	85.07	182.87	317.45	800.51	1678.56
**SSP2-RCP2.6**	0.000001	0.000002	16.07	133.42	404.30	1152.19	2194.19	3604.88	5212.59
**SSP2-RCP1.9**	0.000001	0.000002	14.13	152.65	363.55	1155.53	2792.47	5466.11	7451.19

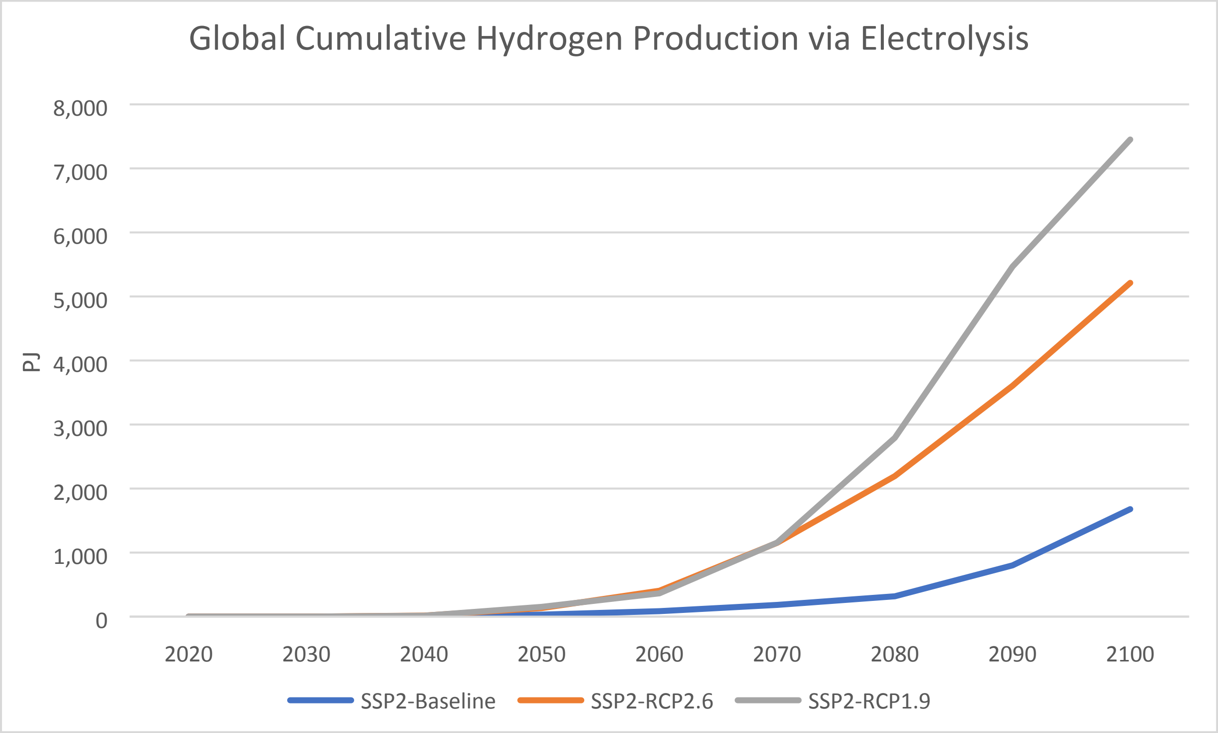

Figure S4. Global cumulative hydrogen production via electrolysis across scenarios.

US power mix

Figure S5 outlines the distinct electricity mix projections for the US across the three scenarios. The *SSP2-Baseline* does not follow a decarbonization target and shows a distinct utilization of natural gas (without CCS) until 2100. The *SSP2-RCP2.6* reaches a net zero GHG emissions economy by 2070, largely driven by the deployment of renewable energy and power generation technologies with CCS. The *SSP2-RCP1.9* achieves an economy-wide decarbonization by 2050 as well as an electricity sector decarbonization by 2035 due to earlier and more aggressive shifts towards low carbon technologies, notably photovoltaics and natural gas with CCS. Coal- and oil-based generation phase-out ten years prior in the stricter mitigation scenario (2040, instead of 2050).

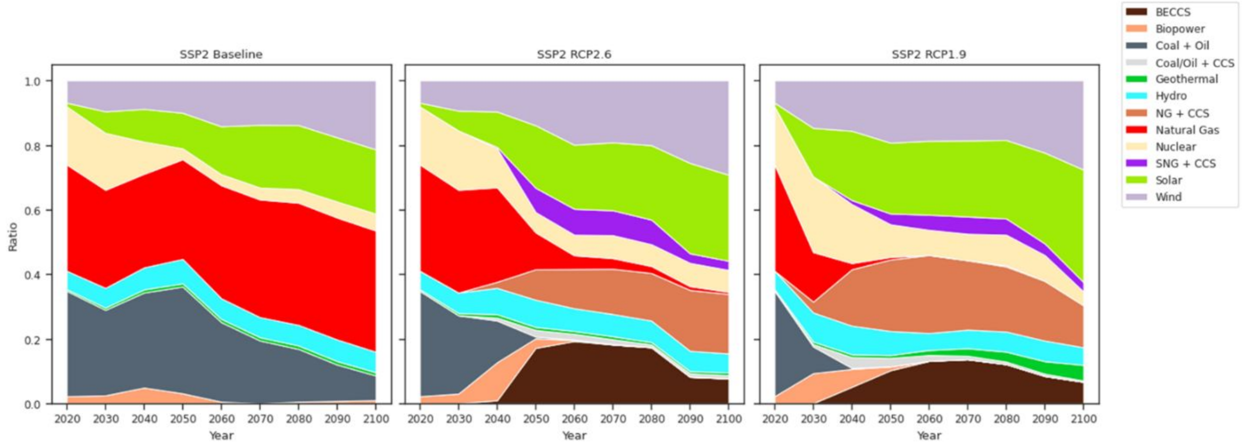

Figure S5. US electricity mix under a baseline, RCP2.6, and RCP1.9 mitigation scenario for SSP2.

Results with TRACI

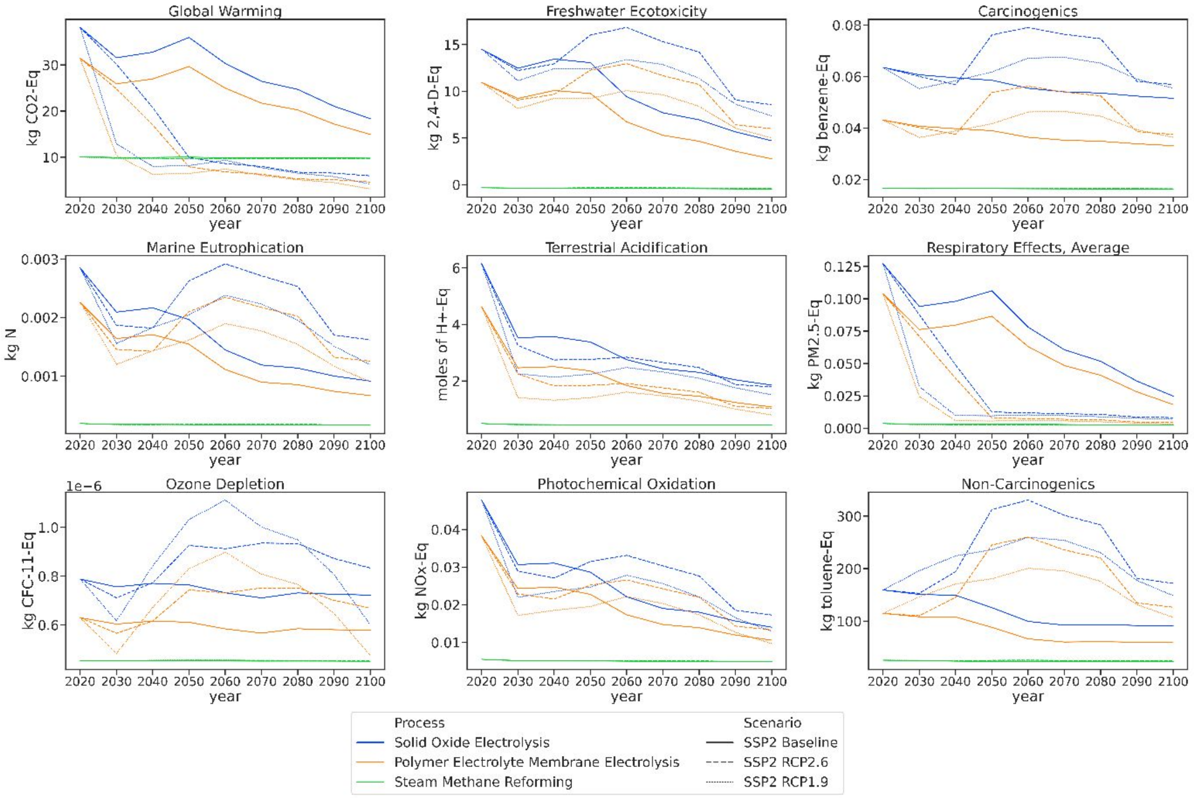

Figure S6. Comparison of all technologies across scenarios under a changing US multi-sector context (background dynamics only) when applying TRACI; effects per kg H_2.

Regional results

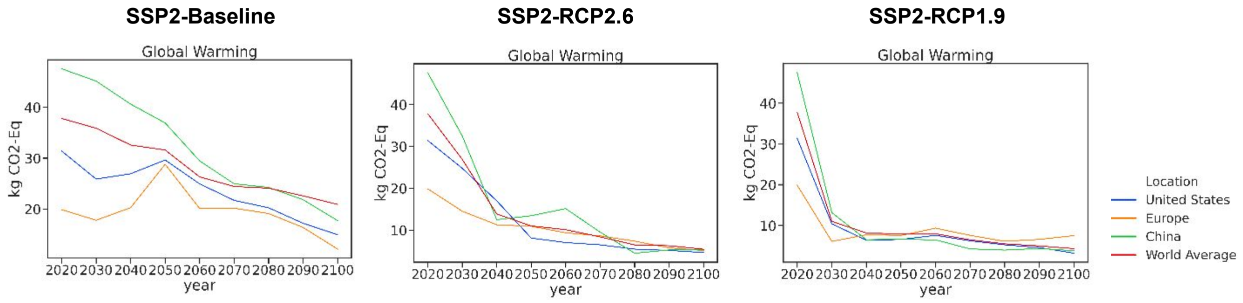

Figure S7. PEME technology impacts across different world regions in comparison to the US; effects per kg H_2.

Comparison to other studies

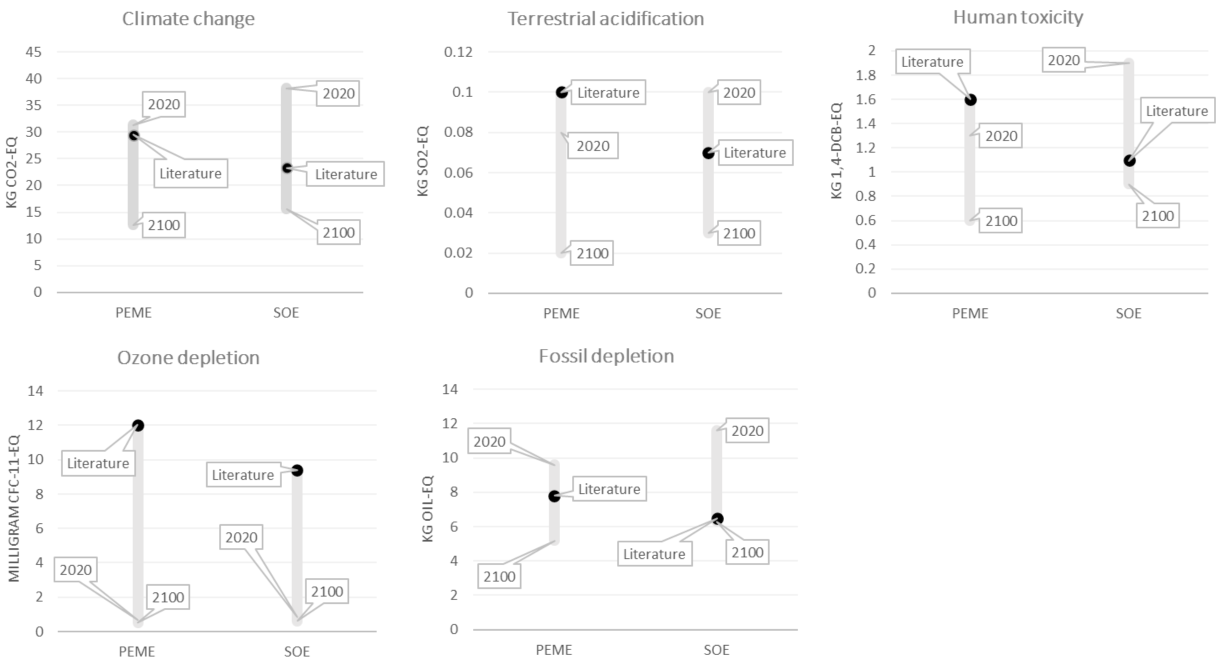

Figure S8. Comparison of SSP2-RCP1.9 results to values by (Mehmeti et al., 2018) and (Häfele et al., 2016) ; effects per kg H_2.

Stochastics

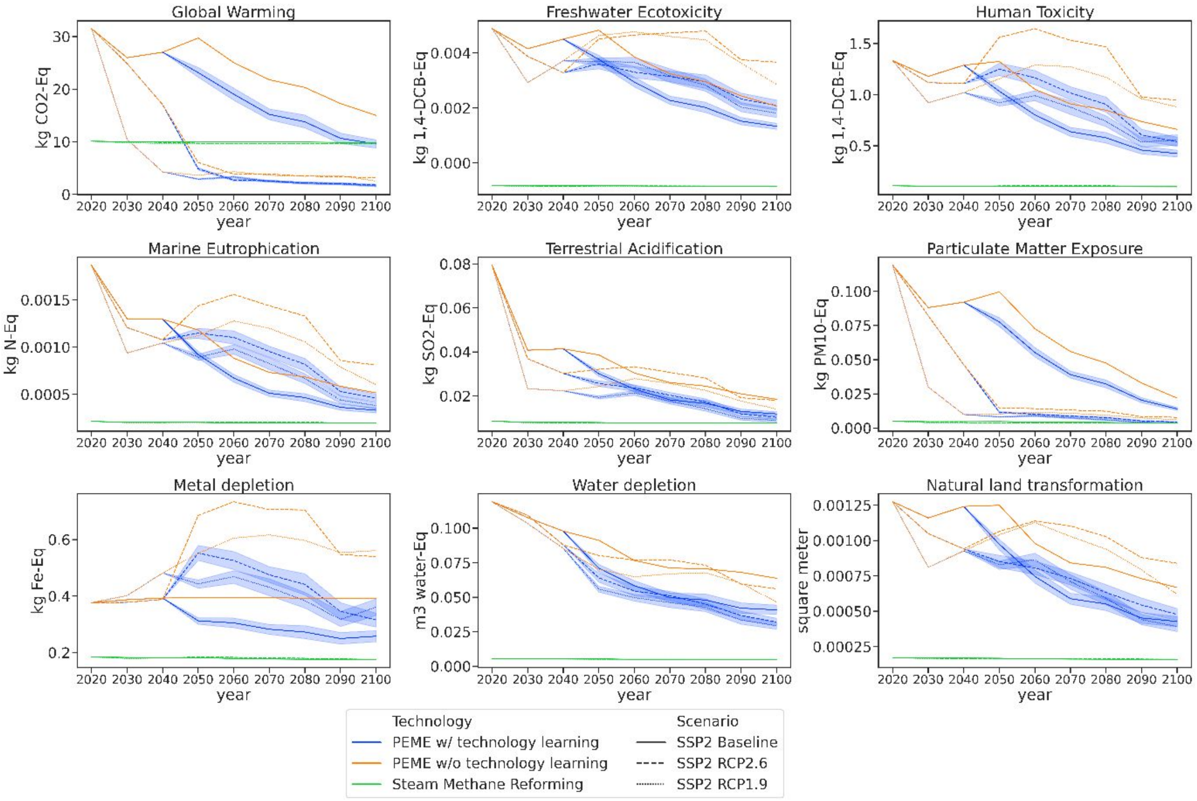

Figure S9. Stochastics analysis for PEME across three scenarios; effects per kg H_2.
